# Machine Learning-Based Personalized Risk Prediction Model for Mortality of Patients Undergoing Mitral Valve Surgery: The PRIME Score

**DOI:** 10.3389/fcvm.2022.866257

**Published:** 2022-04-01

**Authors:** Ning Zhou, Zhili Ji, Fengjuan Li, Bokang Qiao, Rui Lin, Wenxi Jiang, Yuexin Zhu, Yuwei Lin, Kui Zhang, Shuanglei Li, Bin You, Pei Gao, Ran Dong, Yuan Wang, Jie Du

**Affiliations:** ^1^Beijing Anzhen Hospital, Capital Medical University, Beijing, China; ^2^Beijing Institute of Heart, Lung and Blood Vessel Diseases, Beijing Anzhen Hospital, Capital Medical University, Beijing, China; ^3^Department of Cardiac Surgery, Beijing Anzhen Hospital, Capital Medical University, Beijing, China; ^4^Department of Epidemiology and Biostatistics, School of Public Health, Peking University, Beijing, China; ^5^Department of Cardiac Surgery, Chinese People’s Liberation Army General Hospital, Beijing, China; ^6^Peking University Health Science Center, Peking University, Beijing, China; ^7^Key Laboratory of Molecular Cardiovascular Sciences, Ministry of Education, Peking University, Beijing, China

**Keywords:** mitral valve surgery, machine learning, risk stratification, personalized risk prediction, mortality

## Abstract

**Background:**

Mitral valve surgery (MVS) is an effective treatment for mitral valve diseases. There is a lack of reliable personalized risk prediction models for mortality in patients undergoing mitral valve surgery. Our aim was to develop a risk stratification system to predict all-cause mortality in patients after mitral valve surgery.

**Methods:**

Different machine learning models for the prediction of all-cause mortality were trained on a derivation cohort of 1,883 patients undergoing mitral valve surgery [split into a training cohort (70%) and internal validation cohort (30%)] to predict all-cause mortality. Forty-five clinical variables routinely evaluated at discharge were used to train the models. The best performance model (PRIME score) was tested in an externally validated cohort of 220 patients undergoing mitral valve surgery. The model performance was evaluated according to the area under the curve (AUC). Net reclassification improvement (NRI) and integrated discrimination improvement (IDI) were compared with existing risk strategies.

**Results:**

After a median follow-up of 2 years, there were 133 (7.063%) deaths in the derivation cohort and 17 (7.727%) deaths in the validation cohort. The PRIME score showed an AUC of 0.902 (95% confidence interval [CI], 0.849–0.956) in the internal validation cohort and 0.873 (95% CI: 0.769–0.977) in the external validation cohort. In the external validation cohort, the performance of the PRIME score was significantly improved compared with that of the existing EuroSCORE II (NRI = 0.550, [95% CI 0.001–1.099], *P* = 0.049, IDI = 0.485, [95% CI 0.230–0.741], *P* < 0.001).

**Conclusion:**

Machine learning-based model (the PRIME score) that integrate clinical, demographic, imaging, and laboratory features demonstrated superior performance for the prediction of mortality patients after mitral valve surgery compared with the traditional risk model EuroSCORE II.

**Clinical Trial Registration:**

[http://www.clinicaltrials.gov], identifier [NCT05141292].

## Introduction

Mitral valve disease is the most common valve disease, patients with severe valvular disease progress rapidly, leading to heart failure and even life-threatening (1, 2). Although the hemodynamics of patients can be corrected by valve surgery, not everyone benefits equally, and the mortality rate remains high among these patients. Careful evaluation of the risk of mortality plays a fundamental role in clinical management, with important implications for the management of personalized diagnosis and treatment (3).

The guidelines for the management of valvular heart disease recommend that risk prediction models be developed to assess personalized risk for patients undergoing valvular surgery (4, 5). To this aim, several risk prediction models have been developed to assess the risk of death in patients after valve surgery (6–8). Traditional risk scores have been developed and validated in surgical populations, primarily for patients undergoing coronary artery bypass grafting. These algorithms have been shown to overestimate the risk of mortality, particularly in the valvular subgroup, and there are limited data on risk assessment for patients with mitral valve disease (9, 10). Most studies predicted perioperative mortality after surgery (4); however, perioperative adverse events only accounted for a small proportion of the overall deaths, and with the improvement of surgical techniques, the perioperative event rate decreased significantly (11, 12). Therefore, the clinical need for a comprehensive prognostic assessment of patients undergoing mitral valve surgery (MVS) remains unmet.

The existing risk score, EuroSCORE II, is mainly based on clinical characteristics, surgical factors, and imaging features. In addition to these factors, hemodynamic abnormalities caused by valvular disease can lead to a series of complex pathophysiological processes that affect patient outcomes, such as inflammation, left ventricular volume overload, and left ventricular systolic dysfunction, which can be reflected by clinical laboratory indicators (4, 5). Machine learning may be a useful tool for comprehensive and personalized assessment of prognosis.

Current risk prediction models were developed using classic statistical modeling techniques constrained by assumptions such as distribution normality, non-informative or random censoring, and hazard risk linearity. Machine learning methods can overcome these limitations by capturing high-dimensional non-linear relationships among a large number of clinical features (13). The effectiveness of this approach has been demonstrated in several medical applications for cardiovascular diseases. For example, machine learning-based models for mortality in patients treated with cardiac resynchronization therapy and prediction of all-cause mortality in patients with suspected coronary artery disease (14, 15). However, few studies have evaluated the risk of mortality in patients after mitral valve surgery based on machine learning.

Therefore, we sought to develop a machine learning-based risk stratification model integrating clinical, demographic, imaging, and laboratory features to predict mortality in patients undergoing mitral valve surgery. We hypothesized that machine learning can capture the relationship between clinical characteristics and develop a risk stratification system to assess the mortality of patients undergoing mitral valve surgery.

## Materials and Methods

### Data Sets

To develop the machine learning models, we used a derivation cohort of 1,883 adult patients (≥18 years) who underwent mitral valve surgery in the Beijing Anzhen Hospital from January 2019 to December 2019. To assess the performance of the models, we used an external validation cohort that included 220 adult patients in the Chinese People’s Liberation Army General Hospital from January 2019 to December 2019. These patients were obtained from the Registry Study of Biomarkers in Mitral Valve Disease (BIOMS-MVD, NCT05141292).

The study protocol complied with the Declaration of Helsinki and was approved by the Beijing Anzhen Hospital Ethics Review Board, and data were routinely collected from a database of electronic medical records by a multicenter research platform.

**FIGURE 1:**
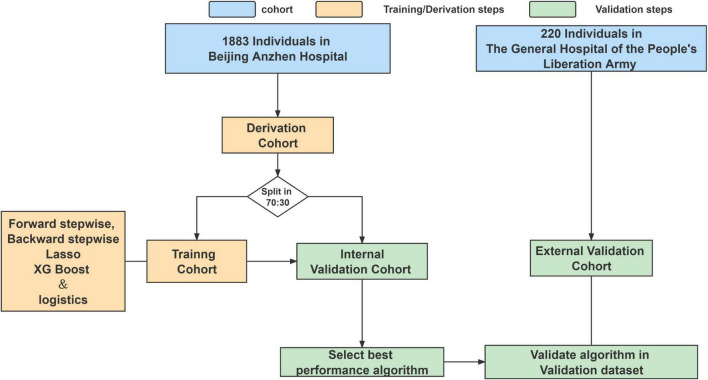
Analysis overview for identifying best-performing risk prediction model.

The patients were diagnosed by echocardiography, and surgery was scheduled according to the 2017 ESC/EACTS Guidelines for the management of valvular heart disease (16). The doctor who performed the operation performs more than 25 mitral valve operations per year, or the surgeon’s unit has surgeons who perform 50 operations per year. We excluded patients who underwent mitral valve surgery at age ≤18 years, did not have full medical records, had >50% study data missing, and the patients after balloon dilation and perivalvular leakage repair who did not undergo cardiopulmonary bypass (Figure 1). The other included surgery is aortic surgery, aortic valve surgery, tricuspid valve repair surgery, radio-frequency ablation, combined coronary artery bypass grafting, ventricular septal repair, and atrial septal repair. Procedures for the inclusion and exclusion of patients are provided in Supplementary Methods 1.

### Outcomes

The endpoint was all-cause mortality. We obtained outcome data from patient visits, medical records, and telephone interviews. The median follow-up time was 2 years, and the cut-off follow-up time point was December 31, 2021. A total of 230 patients were not provided with follow-up information due to lack of contact information or withdrawal.

### Feature Selection and Data Preprocessing

Data quality control was performed before the data analysis. Candidate variables included the patient’s demographic and clinical characteristics, as well as imaging, surgical, and laboratory variables, with a total of 45 daily variables. The imaging and laboratory indexes obtained were the last test before discharge after valvular surgery. Each category of variables is detailed in Supplementary Methods 2. The predictive variables of EuroSCORE II included sex, age, chronic pulmonary disease, extracardiac arteriopathy, neurological dysfunction, previous cardiac surgery, serum creatinine, active endocarditis, critical preoperative state, unstable angina, recent myocardial infarct, pulmonary hypertension, left ventricular ejection fraction (LVEF), emergency, other than isolated CABG, surgery on thoracic aorta, and postinfarct septal rupture.

### Model Development and Validation

The derivation cohort was randomly split into a training cohort and an internal validation cohort with a ratio of 70:30. We used 10 datasets for multiple imputation to handle missing values. The model was trained on each imputed dataset (Missing values are shown in Supplementary Tables 1, 2).

The model was developed by forward stepwise, backward stepwise, Lasso regression, and XGBoost methods to screen variables, and the logistic regression model was used for modeling. Receiver operating characteristic curves were used to estimate model discrimination by calculating the area under the curve (AUC). In the internal validation cohort, the best performance model (PRIME score) was chosen for further evaluation in the 220 external cohort. PRIME score were compared with the traditional model in terms of net reclassification improvement (NRI) and integrated discrimination improvement (IDI). As for the comparison between the developed model and existing risk strategies, the EuroSCORE II is mainly used as the prediction scoring model of mortality (4, 5).

### Statistical Analysis

**FIGURE 2:**
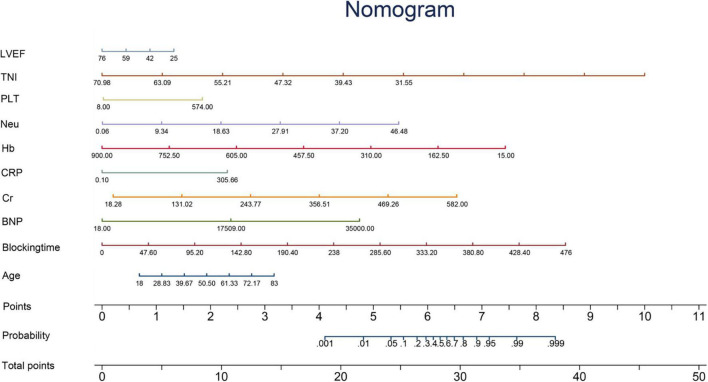
Nomogram. The nomogram is constructed according to the regression coefficient in the model, which is convenient for better clinical application. Blocking time, blocking time of the ascending aorta during surgery; BNP, brain natriuretic peptide; Cr, serum creatinine; CRP, C-reactive protein; Hb, hemoglobin; Neu, neutrophil count; PLT, platelet count; TNI, cardiac troponin I; LVEF, left ventricular ejection fraction.

For continuous variables, normally distributed variables were represented by mean ± standard deviation, and non-normally distributed variables were represented by median (quartile range). Categorical variables were expressed as frequencies or percentages. Two groups of continuous variables were compared using the bilateral independent *t*-test or Wilcoxon test, and categorical variables were compared using the chi-square test or Fisher’s exact test and the Mann–Whitney *U* test. We analyzed the predictor variable effects using the odds ratio (OR) values and beta coefficients in the model. Kaplan–Meier estimates were used to construct survival curves based on all available follow-up data for time-to-event analysis. In addition, a nomogram was developed to predict individual mortality for each patient (Figure 2).

Analyses were performed using Stata 15.1 (Stata Corp, College Station, TX, United States) and R v4.0.3 (R Foundation for Statistical Computing, Vienna, Austria; packages party, MASS, rms, missForest).

## Results

The demographic, clinical, surgical, imaging, and laboratory characteristics of the study population are shown in Table 1. In the derivation cohort, 1,318 patients (70%) split into the training cohort and 565 patients (30%) into the internal validation cohort. In the derivation cohort 133 patients (7.06%) died by the mean median follow-up of 2 years (Table 1). In the external validation cohort, 17 patients (7.13%) died at a median follow-up of 2 years. The mean value of the internal validation cohort EuroSCORE II was 10.758 ± 2.408, and the external validation cohort EuroSCORE II was 4.164 ± 3.039.

**TABLE 1:** Baseline characteristics of patients undergoing mitral valve surgery according to the derived cohort and validation cohort (*n* = 2,103)*.

Variables	Derivation cohort	External validation cohort	*P*-value
		
	(*n* = 1883)	(*n* = 220)	
**Demographic variables**			
Male (*N*, %)	982 (52.2)	113 (51.4)	0.825
Age (Median ± SD)	59 ± 11.37	57.5 ± 13.63	0.060
EuroSCORE II	10 ± 2.37	4 ± 3.04	<0.001
**Clinical variables**			
NYHA			0.001
NYHA = 1 (*N*, %)	3 (0.3)	1 (0.8)	
NYHA = 2 (*N*, %)	435 (40.6)	33 (28.0)	
NYHA = 3 (*N*, %)	560 (52.2)	65 (55.1)	
NYHA = 4 (*N*, %)	74 (6.9)	19 (16.1)	
Smoke (*N*, %)	453 (24.1)	38 (17.3)	0.024
Drinking (*N*, %)	315 (16.7)	46 (20.9)	0.120
Hypertension (*N*, %)	574 (30.5)	49 (22.3)	0.012
Diabetes (*N*, %)	209 (11.1)	15 (6.8)	0.051
Hyperlipidemia (*N*, %)	218 (11.6)	4 (1.8)	<0.001
CAD (*N*, %)	363 (19.3)	46 (20.9)	0.563
Syncope (*N*, %)	31 (1.6)	0	0.009
AF (*N*, %)	797 (42.3)	82 (37.3)	0.150
Pre-MI (*N*, %)	78 (4.1)	1 (0.5)	0.001
Pre-surgery (*N*, %)	133 (7.1)	21 (9.5)	0.181
Pre-valve surgery (*N*, %)	88 (4.7)	19 (8.6)	0.019
Renal insufficiency (*N*, %)	58 (3.1)	9 (4.1)	0.436
Infect endocarditis (*N*, %)	58 (3.1)	19 (8.6)	<0.001
Central nervous (*N*, %)	136 (7.2)	24 (10.9)	0.051
Lung disease (*N*, %)	53 (2.8)	12 (5.5)	0.050
Peripheral vd (*N*, %)	18 (1)	0	0.136
**Imaging variables**			
LA (Median ± SD)	40 ± 7.39	37 ± 9.34	<0.001
VST (Median ± SD)	10 ± 1.94	11 ± 1.95	<0.001
LVEDD (Median ± SD)	47 ± 6.46	45 ± 335.1	<0.001
Lv thickness (Median ± SD)	10 ± 1.56	11 ± 4.70	<0.001
LVEF (Median ± SD)	57 ± 8.07	57 ± 9.61	0.376
Tr area (Median ± SD)	1 ± 2.39	0 ± 0.95	0.496
PG (Median ± SD)	15 ± 9.13	15 ± 111.98	0.089
**Laboratory variables**			
CKMB (Median ± SD)	35 ± 41.03	4.12 ± 16.91	<0.001
TNI (Median ± SD)	3.09 ± 6.00	0.635 ± 1.20	<0.001
CRP (Median ± SD)	41.79 ± 58.57	4.30 ± 4.61	<0.001
Cr (Median ± SD)	66.4 ± 44.28	75.4 ± 87.59	<0.001
Alb (Median ± SD)	36.2 ± 6.46	38.3 ± 4.98	<0.001
Hb (Median ± SD)	99 ± 23.98	103 ± 56.17	0.004
Lym (Median ± SD)	1.45 ± 1.39	0.12 ± 0.07	<0.001
Neu (Median ± SD)	8.09 ± 4.57	0.79 ± 0.11	<0.001
PLT (Median ± SD)	171 ± 83.1	145 ± 82.57	<0.001
BNP (Median ± SD)	234 ± 590.10	1541 ± 5432	<0.001
**Surgical variables**			
Combined aortic surgery (*N*, %)	1 (0.1)	0	0.062
Combined avr (*N*, %)	388 (20.6)	35 (15.9)	0.100
Combined tvp (*N*, %)	1098 (58.3)	119 (54.1)	0.230
Combined ra (*N*, %)	511 (27.1)	30 (13.6)	<0.001
Combined cabg (*N*, %)	302 (16.0)	21 (9.5)	0.011
Combined asd (*N*, %)	35 (1.9)	6 (2.7)	0.403
Combined vsd (*N*, %)	8 (0.4)	2 (0.9)	0.282
Cpb time (Median ± SD)	138 ± 57.47	140 ± 57.70	0.205
Blocking time (Median ± SD)	97 ± 34.64	108.5 ± 52.85	<0.001

*For continuous variables, non-normally distributed variables are expressed as median [interquartile ranges (IQRs)] and normally distributed variables are expressed as means [standard deviation (SD)]. Categorical variables are expressed in N (%). P < 0.05 was considered to be statistically significant. NYHA, New York Heart Association classification; CAD, coronary heart disease; AF, atrial fibrillation; Pre-MI, previous myocardial infarction; Pre-surgery, previous surgery; Pre-valve surgery, previous valve surgery; Central nervous, previous central nervous system disease; LA, left atrial; VST, ventricular septal thickness; LVEDD, left ventricular end diastolic volume; Lv thickness, left ventricular wall thickness; LVEF, left ventricular ejection fraction; Tr area, tricuspid regurgitation area; PG, cross valve pressure gradient; CKMB, creatine kinase MB; TNI, cardiac troponin I; CRP, C-reactive protein; Cr, serum creatinine; Alb, serum albumin; Hb, hemoglobin; Lym, lymphocyte count; Neu, neutrophil count; PLT, platelet count; BNP, brain natriuretic peptide; avr, aortic valve surgery; tvp, tricuspid valve repair surgery; ra, radiofrequency ablation; cabg, coronary artery bypass grafting; asd, atrial septal repair; vsd, ventricular septal repair; Cpb time, cardiopulmonary bypass time; Blocking time, blocking time of the ascending aorta during surgery.

Risk prediction models were developed by XGBoost, forward stepwise, backward stepwise, and Lasso regression in the training cohort. The beta coefficient and OR value of the different prediction models are shown in Table 2 and Supplementary Tables 3–5. The performance of the different models in the internal validation cohort is shown in Supplementary Figure 1.

**TABLE 2:** Beta coefficients and odds ratios of the PRIME Score.

Variables	Odds Ratio	[95% CI]		β -coefficient	*P*-value
Age	1.01902	0.9953273	1.043277	0.0188414	0.651
Blocking time	1.009855	1.003684	1.016063	0.0098064	0.001
BNP	1.000385	1.000059	1.000712	0.0003853	0.056
Cr	1.007701	1.003684	1.011735	0.007672	0.008
CRP	1.004806	1.000548	1.009082	0.0047945	0.047
Hb	0.9803747	0.9675595	0.9933597	−0.0198204	0.646
Neu	1.067973	1.021857	1.116171	0.0657629	0.103
PLT	1.000413	0.9971895	1.003647	0.0004128	0.430
TNI	0.9950825	0.9613893	1.029957	−0.0049296	0.022
LVEF	0.9712531	0.9474292	0.9956762	−0.0291682	0.673

*Blocking time, blocking time of the ascending aorta during surgery; BNP, brain natriuretic peptide; Cr, serum creatinine; CRP, C-reactive protein; Hb, hemoglobin; Neu, neutrophil count; PLT, platelet count; TNI, cardiac troponin I; LVEF, left ventricular ejection fraction.*

**FIGURE 3:**
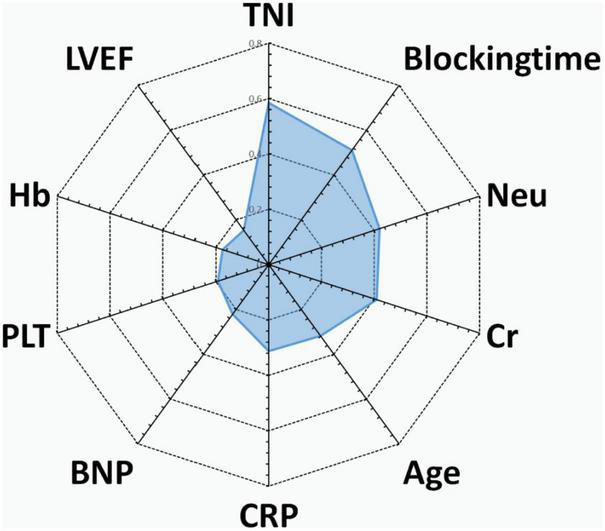
Radar chart of the 10 most important predictors of poor prognosis screened by the PRIME score. For each important predictor, standardized beta coefficients of overall population are shown. Blocking time, blocking time of the ascending aorta during surgery; BNP, brain natriuretic peptide; Cr, serum creatinine; CRP, C-reactive protein; Hb, hemoglobin; Neu, neutrophil count; PLT, platelet count; TNI, cardiac troponin I; LVEF, left ventricular ejection fraction.

Predictive variables for the PRIME score included age, blocking time (The blocking time is time delta between the two time records of ascending aorta occlusion and re-perfusion simultaneously for each patient), creatinine, left ventricular ejection fraction (LVEF), neutrophils, C-reactive protein (CRP), Cardiac Troponin I (TNI), hemoglobin, B-type natriuretic peptide (BNP) and platelets. The beta coefficient and OR value of the model are shown in Table 2. Leading predictors varied according to the study outcomes of the overall population (Figure 3). A nomogram was constructed according to the regression coefficients in the model for clinical use, such as in information sharing and decision making for both clinicians and patients (Figure 2).

**FIGURE 4:**
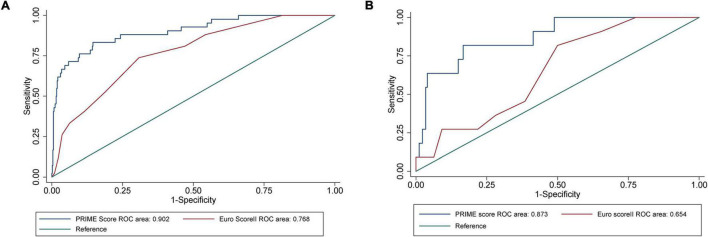
Performance evaluation of internal validation cohort and external verification cohort on the PRIME score and verification of EuroSCORE II. **(A)** The area under the receiver operating characteristic (ROC) curves showed that the area under the curve (AUC) of all-cause death in the internal validation cohort of the PRIME score was 0.9021 (95% confidence interval [CI]: 0.8487–0.9555), which was better than that of EuroSCORE II (*P* = 0.0011). **(B)** The area under the ROC curve showed that the prime score with AUC of 0.8730 (95% CI: 0.7690–0.9770) in the external validation cohort was also better than EuroSCORE II (*P* = 0.0062).

The AUC of EuroSCORE II was 0.768 (95% CI [confidence interval]: 0.699–0.838) in the internal validation cohort and 0.654 (95% CI: 0.514–0.795) in the external validation cohort. The PRIME score achieved an AUC of 0.902 (95% CI: 0.849–0.956) in the internal validation cohort, which was better than the EuroSCORE II (*P* = 0.001). When applied to the external validation cohort, the AUC was 0.873 (95% CI: 0.769–0.977), which was also better than the EuroSCORE II (*P* = 0.006) (Figure 4). The mortality were assessed in four subgroups quartile by EuroSCORE II (shown in Supplementary Figure 2). The PRIME score demonstrated robust performance in different risk subgroup patients divided by EuroSCORE II, highlighting its generalizability across different risk settings. In addition, compared with the EuroSCORE II, the performance of the model in reclassification and recognition was significantly improved in the externally validated cohort (NRI = 0.550, [95% CI 0.001–1.099], *P* = 0.049, IDI = 0.485, [95% CI 0.230–0.741], *P* < 0.001) (Table 3).

**TABLE 3:** Improved model performance over the EuroSCORE II.

Statistic	Estimate	[95% CI]		*P*-value	Statistic	Estimate	[95% CI]		*P*-value
NRI (Controls)	–0.086	−0.210	0.038	0.173	IDI (Controls)	−0.018	−0.046	0.010	0.210
NRI (Cases)	0.636	0.102	1.171	0.020	IDI (Cases)	0.503	0.249	0.757	<0.001
NRI (Overall)	0.550	0.001	1.099	0.049	IDI (Overall)	0.485	0.230	0.741	<0.001

*CI, confidence interval; NRI, net reclassification improvement; IDI, integrated discrimination improvement. Performance improvement compared with EuroSCORE II.*

**FIGURE 5:**
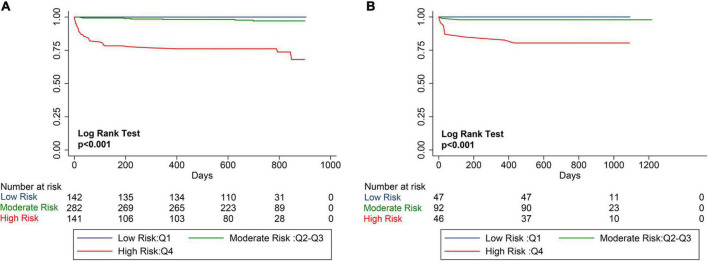
Kaplan–Meier Estimates of mortality for internal validation cohort and external verification cohort. The survival rate was observed using the Kaplan–Meier curve and compared using the log-rank test. **(A)** Kaplan–Meier estimated the survival rate of the internal validation cohort using the PRIME score. **(B)** Kaplan–Meier estimated the survival rate of the external validation cohort using the PRIME score. As shown by the Kaplan–Meier curve, the two cohorts showed significant differences in mortality among the three risk score groups (*P* < 0.0001).

The mean PRIME risk score of the internal and external validation cohorts were 0.069 ± 0.122 and 0.124 ± 0.244, respectively. The PRIME score effectively stratifies the risk of all-cause mortality into low-risk, medium-risk, and high-risk subsets. As shown in the Kaplan–Meier curves, the internal and external validation cohorts showed significant differences in mortality among the three risk assessment subgroups, significant differences in 2-year mortality (*P* < 0.001), and a significant increase in mortality rates was observed in the high-risk group (Figure 5).

## Discussion

In this study, we used data from 2,103 patients discharged after mitral valve surgery to develop and validate a machine learning-based risk prediction system, using the PRIME score, to predict the risk of mortality. We identified predictive variables from routine clinical data to generate a risk prediction model. We found that the PRIME score presented excellent discriminative abilities for the prediction of all-cause mortality. Compared with pre-existing risk score, the PRIME score had improved discriminatory power and predictive range for all-cause mortality. In addition, the PRIME score was able to identify patients with a significantly increased risk of all-cause mortality throughout the follow-up period.

Simultaneous interpretation of the myriad risk predictors for individual patients is a challenge for clinicians. The complexity of the assessment is increased by the large number of clinical variables that need to be considered in relation to mortality, which makes it more difficult for clinicians to draw overall conclusions about the risks for individual patients. In addition, the potential effects of complex and hidden interactions between several weaker predictors are often overlooked. In this study, we demonstrate that machine learning can overcome these challenges by utilizing complex high-level interactions. We aimed to develop a accurate risk stratification system integrating high-dimensional and features to predict mortality. XGBoost, lasso and stepwise regression are all widely used variable selection methods based on different mathematical theories. XGBoost algorithm was employed due to its state-of-the-art accuracy and interpretability (17). LASSO was used for it is a popular method for regression with high-dimensional predictors. This approach has been extended and broadly applied for survival analysis with high-dimensional data (18). We used the methods to filter the predictive variables. In addition, logistic regression was used for risk model building to solve the binary classification problems in this study (19). Therefore, we used the methods for selecting the variables and constructing the risk model, and chose the best performance model named the PRIME score.

The PRIME score has improved the ability and predictive range of all-cause mortality due to the explainable predictive variables that reflect individual pathophysiological factors. Age factors have an impact on the postoperative prognosis, such as decreased physiological reserve, resistance to stressors, and increased vulnerability (20). Second, chronic renal insufficiency causes myocardial damage caused by cardiac structure and function changes, which seriously affects the patient’s prognosis. Patients should reduce creatinine levels by reducing the use of contrast agents, using renal protective measures, and more aggressive intermittent renal replacement therapy to protect kidney function (21). Third, the LVEF is related to the contractility of the myocardium. Patients with myocardial systolic dysfunction should control the volume load and take drugs that can improve the myocardial systolic function (22, 23). The aspects mentioned above are all mentioned in traditional scoring, while the new variables include surgical procedures such as blocking time and pathological indicators reflecting different pathophysiological states. Fourth, the blocking time of the ascending aorta during surgery has a great influence on myocardial injury in patients undergoing valve surgery, and it is important to control the aortic clamp for valve surgery (24). Fifth, laboratory indicators reflecting pathophysiological states lead to different postoperative outcomes in individual patients (25). With the decrease of hemoglobin in patients, the blood oxygen-carrying capacity is reduced, and the myocardium requires a larger cardiac output to compensate and maintain its function, resulting in ventricular remodeling and dysfunction (26, 27). The rapid or continuous decrease in platelet count in a short period of time often indicates the possibility of acute platelet dysfunction and poor prognosis (28). Bleeding should be controlled as much as possible during surgery. If a decrease in hemoglobin and platelet levels is found, the etiology should be actively sought and timely intervention should be performed. The levels of neutrophils and C-reactive protein reflect the inflammatory state of the body and the degree of damage to myocardial tissue, which is a fast and simple method to evaluate the inflammatory state and a predictor of cardiovascular risk. Monitoring and controlling inflammation in the diagnosis and treatment of valve patients is crucial for patient prognosis (29). BNP reflects the volumetric load of the left ventricle; patients with elevated BNP levels should control fluid intake and use diuretics according to symptoms to reduce water and sodium retention, which can exacerbate heart failure and lead to poor prognosis (30). Increased troponin I (TnI) levels are associated with a higher risk of death, and patients with high TnI levels should shorten the follow-up period and have regular observation (31).

In summary, the predictive variables included in the PRIME score reflect the combined effects of the system pathological state. With respect to the weights of variables and their contributions to the model, it can be inferred that variables with relatively high weights reflect inherent pathophysiology and are in good agreement with clinical outcomes.

### Strengths

The main advantage of our study, is that compared with the existing risk strategy EuroSCORE II, the PRIME score uses fewer predictive variables, can comprehensively combine demographic characteristics, complications, surgical, and pathophysiological factors, better predict mortality in patients undergoing mitral valve surgery, and may reduce the risk of death by monitoring and controlling these risk factors.

### Limitations

This study had several limitations. First, the data used in this study were from two central cohorts, and although the PRIME score was validated in external cohorts, further exploration should be provided in other less controlled settings. Second, the weights of some of the risk factors considered in the model may change over time, and the follow-up period should be extended for validation. Third, this study was retrospective and could be performed prospectively. Finally, our scoring requires manual input and calculation steps, such as being linked to an electronic medical record system to automatically calculate the risk score; avoiding the process of manual calculation and potentially increasing the use of the model in clinical practice could further improve this scoring system.

## Conclusion

Using daily clinical variables, we established and validated a machine-learning-based personalized risk prediction model for the mortality of patients undergoing mitral valve surgery. This study showed that the machine learning-based method is feasible and effective, with potentially important implications for the management of patients.

## Data Availability Statement

The original contributions presented in the study are included in the article/Supplementary Material, further inquiries can be directed to the corresponding author/s.

## Ethics Statement

Written informed consent was obtained from the individual(s) for the publication of any potentially identifiable images or data included in this article.

## Author Contributions

NZ conceptualized the study, carried out the analyses, and drafted the initial manuscript. ZJ participated in the establishment and judgment of the multi-center cohort, made suggestions for medical records of patients, and revised the manuscript in this study. YW and RD conceptualized the study, supervised the analyses, and reviewed and revised the manuscript. JD conceptualized the study, supervised the analyses, reviewed and revised the manuscript, and was responsible for the administration of the BIOMS-MVD registry study. YL and YZ conducted data collection and statistical analysis. PG supervised the analyses and reviewed the manuscript. RL, KZ, SL, and WJ collected the data and conducted the literature search. FL and BQ conceptualized the study and reviewed the manuscript. All authors were involved in the manuscript review and agreed to be accountable for the content of the work.

## Conflict of Interest

The authors declare that the research was conducted in the absence of any commercial or financial relationships that could be construed as a potential conflict of interest.

## Publisher’s Note

All claims expressed in this article are solely those of the authors and do not necessarily represent those of their affiliated organizations, or those of the publisher, the editors and the reviewers. Any product that may be evaluated in this article, or claim that may be made by its manufacturer, is not guaranteed or endorsed by the publisher.
