# Supplementary material for: Machine Learning-Based Personalized Risk Prediction Model for Mortality of Patients Undergoing Mitral Valve Surgery: The PRIME Score
Source: Front Cardiovasc Med. 2022 Apr 1;9:866257. doi: 10.3389/fcvm.2022.866257 (PMC9010531; doi:10.3389/fcvm.2022.866257)
Supplement: Supplementary file 1 [file Data_Sheet_1.pdf]

Supplementary Material

Supplementary Method 1

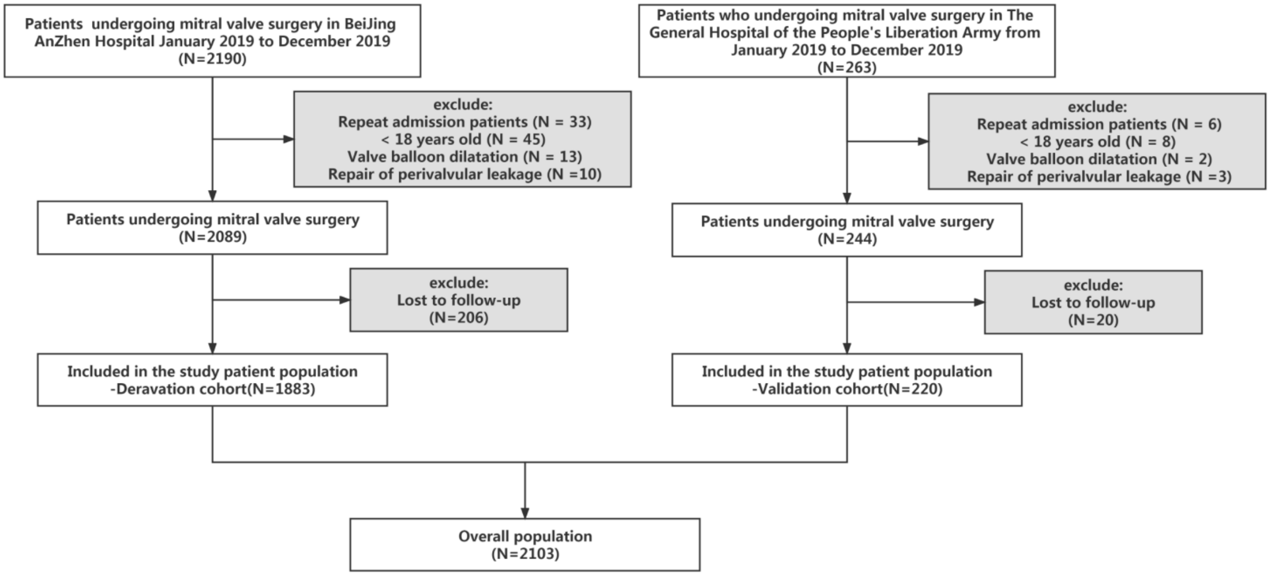

Supplementary Method 1. Flow chart for inclusion and exclusion of patients.

Step 1: Patients who underwent mitral valve surgery in Beijing Anzhen Hospital and Beijing People's Liberation Army General Hospital from January 2019 to December 2019; Step 2: Excluded patients included those who were admitted repeatedly or were of <18 years of age; patients with valve balloon dilatation; patients with perivalvular leakage repair; Step 3: Lost patients were excluded.

Supplementary Methods 2.

1 Candidate variables and definitions

2 (1) Demographic variables: sex, age, Euro Score II score

3 (2) Clinical variables: New York Heart Association classification (NYHA), smoking history, drinking history, hypertension, diabetes, hyperlipidemia, coronary heart disease(CAD), syncope, atrial fibrillation(AF), previous myocardial infarction(Pre-MI), previous surgery(Pre-surgery), previous valve surgery(Pre-valvesurgery), infective endocarditis, previous central nervous system disease(Centralnervous) and peripheral vascular disease.

4 (3) Imaging variables: left atrial (LA) anterior posterior diameter, ventricular septal thickness (VST), left ventricular end diastolic volume (LVEDD), left ventricular wall thickness (Lv thickness), left ventricular ejection fraction (LVEF), tricuspid regurgitation area (Tr area), and cross valve pressure gradient(PG).

5 (4) Laboratory variables: creatine kinase MB (CKMB), cardiac troponin I (TnI), C-reactive protein (CRP), serum creatinine (Cr), serum albumin (Alb), hemoglobin (Hb), lymphocyte count (Lym), neutrophil count (Neu), platelet (PLT), and brain natriuretic peptide (BNP).

6 (5) Surgical related variables included combined aortic surgery, combined aortic valve surgery(avr), combined tricuspid valve repair surgery (tvp), combined radiofrequency ablation (ra), combined coronary artery bypass grafting (cabg), combined ventricular septal repair (vsd), combined atrial septal repair (asd), cardiopulmonary bypass time (cpb time), and blocking time of the ascending aorta during surgery (blocking time).

Supplementary Table 1. Missing value for derivation cohort

Variables	Missing	Total	Percent
**NYHA**	811	1,883	43.07
**Cpb time**	41	1,883	2.18
**Blocking time**	36	1,883	1.91
**CKMB**	40	1,883	2.12
**TNI**	44	1,883	2.34
**CRP**	458	1,883	24.32
**Cr**	24	1,883	1.27
**Alb**	25	1,883	1.33
**Hb**	17	1,883	0.9
**Lym**	17	1,883	0.9
**Neu**	17	1,883	0.9
**PLT**	19	1,883	1.01
**BNP**	474	1,883	25.17
**LA**	361	1,883	19.17
**VST**	491	1,883	26.08
**LVEDD**	223	1,883	11.84
**LV thickness**	506	1,883	26.87
**LVEF**	230	1,883	12.21
**Tr area**	762	1,883	40.47

NYHA, New York Heart Association classification; Cpb time, cardiopulmonary bypass time; Blocking time, blocking time of the ascending aorta during surgery. CKMB, creatine kinase MB; TNI, cardiac troponin I; CRP, C-reactive protein; Cr, serum creatinine; Alb, albumin; Hb, hemoglobin; Lym, lymphocyte; Neu, neutrophil; PLT, platelet; BNP, brain natriuretic peptide; LA, left atrial; VST, ventricular septal thickness; LVEDD, left ventricular end diastolic volume; LV, left ventricular; LVEF, left ventricular ejection fraction; Tr area, tricuspid regurgitation area.

Supplementary Table 2. Missing values for validation cohort

Variables	Missing	Total	Percent
**NYHA**	102	220	46.36
**LA**	26	220	11.82
**LVEDD**	26	220	11.82
**VST**	32	220	14.55
**LV thickness**	32	220	14.55
**LVEF**	26	220	11.82
**Tr area**	26	220	11.82
**Alb**	1	220	0.45
**Cr**	1	220	0.45
**TNI**	9	220	4.09
**CKMB**	8	220	3.64
**BNP**	1	220	0.45
**Hb**	1	220	0.45
**PLT**	1	220	0.45
**Lym**	1	220	0.45
**Neu**	1	220	0.45
**CRP**	2	220	0.91

NYHA, New York Heart Association classification; LA, left atrial;; LVEDD, left ventricular end diastolic volume; VST, ventricular septal thickness; LVEF, left ventricular ejection fraction; Tr area, tricuspid regurgitation area; Alb, albumin; Cr, creatinine; TNI, cardiac troponin I; CKMB, creatine kinase MB; BNP, brain natriuretic peptide; Hb, hemoglobin; PLT, platelet; Lym, lymphocyte; Neu, neutrophil; CRP, C-reactive protein.

Supplementary Table 3. Beta coefficients and odds ratios of Forward Step Model

Variables	Odds Ratio	[95% Confidence Interval]		β-coefficient
**Blocking time**	1.011767	1.003067	1.020544	0.0093287
**Central nervous**	3.212922	1.265785	8.155305	0.0004429
**Lung disease**	2.147308	0.5220882	8.831707	1.147955
**BNP**	1.000477	1.000086	1.000867	0.0004429
**Cr**	1.007369	1.001985	1.012781	0.0081394
**Hb**	1.001007	0.9866801	1.015543	-0.0171007
**Neu**	1.051025	0.9837298	1.122924	0.0649575
**LVEF**	0.9839868	0.9439471	1.025725	-0.0302272
**Pre-surgery**	1.997156	0.6618533	6.026462	0.8590371
**CRP**	1.005784	1.000724	1.010869	0.0048863

Blocking time, blocking time of the ascending aorta during surgery; Central nervous, previous central nervous system disease; BNP, brain natriuretic peptide; Cr, serum creatinine; Hb, hemoglobin; Neu, neutrophil count; LVEF, left ventricular ejection fraction; Pre-surgery, previous surgery; CRP, C-reactive protein.

Supplementary Table 4. Beta coefficients and odds ratios of Backward stepwise Model

Variables	Odds Ratio	[95% Confidence Interval]		β-coefficient
**Blocking time**	1.011767	1.003067	1.020544	0.0093287
**Central nervous**	3.212922	1.265785	8.155305	0.0004429
**Lung disease**	2.147308	0.5220882	8.831707	1.147955
**BNP**	1.000477	1.000086	1.000867	0.0004429
**Cr**	1.007369	1.001985	1.012781	0.0081394
**Hb**	1.001007	0.9866801	1.015543	-0.0171007
**Neu**	1.051025	0.9837298	1.122924	0.0649575
**LVEF**	0.9839868	0.9439471	1.025725	-0.0302272
**Pre-surgery**	1.997156	0.6618533	6.026462	0.8590371
**CRP**	1.005784	1.000724	1.010869	0.0048863

Blocking time, blocking time of the ascending aorta during surgery; Central nervous, previous central nervous system disease; BNP, brain natriuretic peptide; Cr, serum creatinine; Hb, hemoglobin; Neu, neutrophil count; LVEF, left ventricular ejection fraction; Pre-surgery, previous surgery; CRP, C-reactive protein.

Supplementary Table 5. Beta coefficients and odds ratios of Lasso Model

Variables	Odds Ratio	[95% Confidence Interval]		β-coefficient
**Age**	1.009701	0.9743792	1.046304	0.0140125
**Blocking time**	1.011615	1.00295	1.020354	0.0089859
**Central nervous**	3.245967	1.289196	8.172768	0.9337605
**Lung disease**	2.003685	0.4804129	8.356885	1.013468
**BNP**	1.000525	1.000143	1.000907	0.0004637
**Cr**	1.007375	1.001904	1.012876	0.0085259
**CRP**	1.005619	1.000584	1.010679	0.0049192
**Hb**	0.9995798	0.9855241	1.013836	-0.0181859
**LVEF**	0.9850293	0.9449866	1.026769	-0.0303526
**Pre-surgery**	1.9659	0.6465589	5.977435	0.8707946

Blocking time, blocking time of the ascending aorta during surgery; Central nervous, previous central nervous system disease; BNP, brain natriuretic peptide; Cr, serum creatinine; Hb, hemoglobin; CRP, C-reactive protein; Hb, hemoglobin; LVEF, left ventricular ejection fraction; Pre-surgery, previous surgery.

Supplementary Figure 1. The performance of different models of internal validation cohort

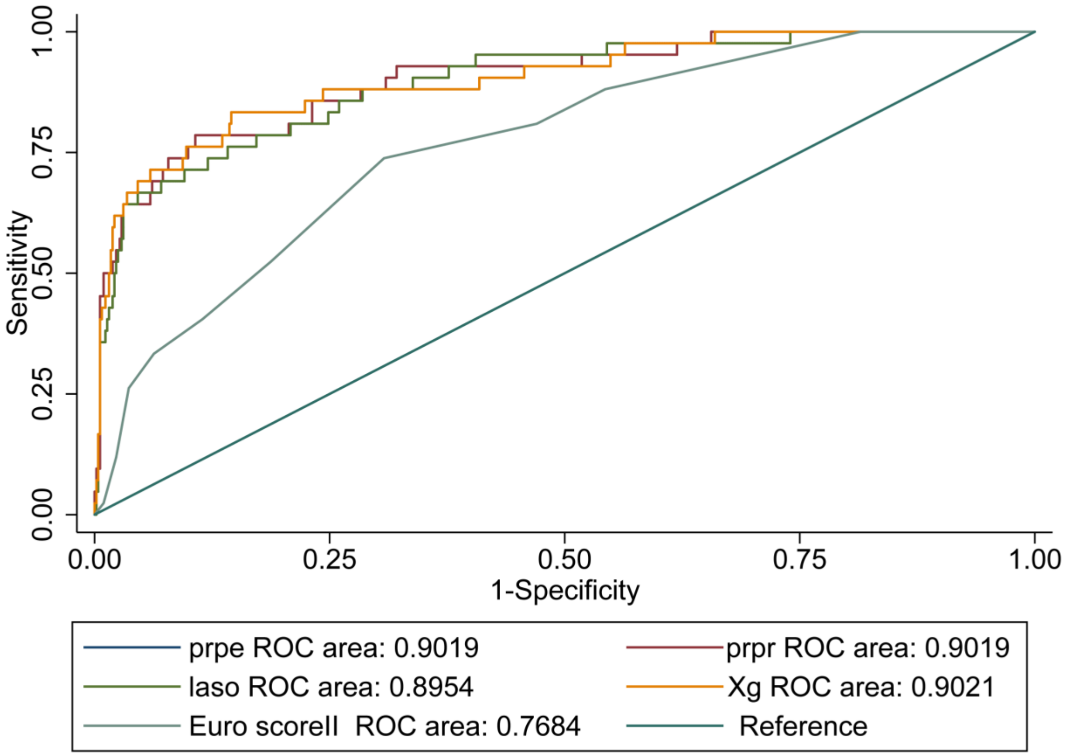

Supplementary Figure 1. Comparing the area under the receiver operating characteristic curve of each model shows that in the internal validation cohort, XG boost model shows the best performance in predicting postoperative adverse events selected as the prime score. prpe, forward stepwise model; prpr, backward stepwise model; laso, Lasso regression model; Xg, XG Boost model.

Supplementary Figure 2.The performance of the PRIME score in different risk subgroup patients divided by Euro score II.

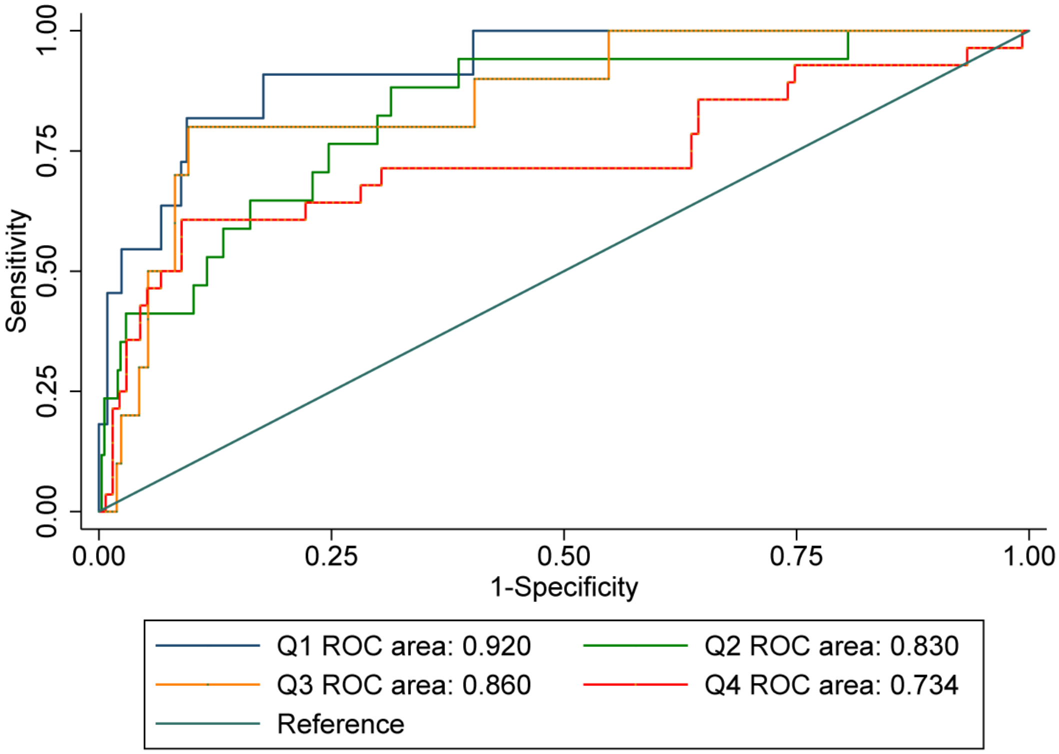

Supplementary Figure 2. Comparing the area under the receiver operating characteristic curve of the PRIME score in different risk subgroup patients divided by Euro score II in overall population. The AUCs in the subgroups were consistent with each other (p = 0.076).
